# Bradycardia and Syncope in a Patient Presenting With Loperamide Abuse

**DOI:** 10.7759/cureus.2599

**Published:** 2018-05-09

**Authors:** Pooja Palkar, Dipti Kothari

**Affiliations:** 1 Psychiatry, Nassau University Medical Center, East Meadow, USA; 2 Medicine, Nassau University Medical Center, East Meadow, USA

**Keywords:** loperamide, abuse, bradycardia, syncope, toxicity

## Abstract

Loperamide is an antidiarrheal agent available as an inexpensive over-the-counter (OTC) medication. In general, it is considered to be safe, but lately, loperamide drug abuse has been reported due to its opioid properties. When used in high doses, several harmful effects including cardiotoxicity, central nervous system (CNS) and respiratory depression have been reported. This prompted the FDA to release a warning in 2016 regarding the arrhythmogenic potential of loperamide. We present a case of a 32-year-old male with a history of polysubstance abuse who presented to the emergency department (ED) requesting “detoxification” from loperamide. The patient complained of opiate withdrawal symptoms including chills, nausea, vomiting, constipation, and abdominal cramps thought to be secondary to the abuse of loperamide. He was found to have right bundle branch block (RBBB) and bradycardia with a heart rate (HR) of 51 beats per min (bpm). He also reported an unexplained syncopal episode, one day prior to visiting the ED. In the current case report, we discuss loperamide abuse, its harmful effects, and management.

## Introduction

Loperamide is an opioid receptor agonist and acts on peripheral mu receptors in the gastrointestinal tract. It works as an antidiarrheal by slowing down movement of the gut and decreasing the frequency of bowel movements. It is used to treat non-infectious diarrhea and commonly used to treat diarrhea due to opioid withdrawal. It has been proven both effective and safe when used at FDA-approved doses (a maximum 16 mg/day for prescription products and 8 mg/day for over-the-counter (OTC) products) [1-2]. Supratherapeutic doses of loperamide have been associated with QTc prolongation and cardiac dysrhythmias. Since dissemination of its opioid properties in online recreational drug use forums, poison control centers have noticed, since 2014, a doubling in referrals for loperamide overdose [3-4]. An accidental and deliberate overdose of loperamide has been associated with opioid-like euphoria [5-6]. In fact, loperamide was, at one time, a schedule V drug due to its opioid properties [4]. Subsequent studies indicated low risk of physical dependence and abuse with the drug and so it was removed from scheduling. However, with increasing abuse, there has been a concomitant increase in the number of reports of toxicity, particularly cardiac manifestations [2-3]. This prompted the FDA to release a warning in 2016 regarding the arrhythmogenic potential of loperamide [7] and subsequently, in January 2018, the FDA limited the packaging for loperamide to encourage safe use [8]. Loperamide is sometimes referred to as “poor man’s methadone” because of its opioid effects, nonprescription status, and low cost.

## Case presentation

We present a case report of a 32-year-male with a past medical history of polysubstance abuse, anxiety, depression, and ADHD who presented to the ED requesting “detoxification” from loperamide. The patient reported that he has been consuming at least 50 to 60 pills of loperamide (2 mg tablets) daily for three years. He started using loperamide three years ago for diarrhea to attenuate heroin and other opiate withdrawal symptoms. Initially, it helped him with diarrhea and over the period of the next few months, he started using the medication excessively to attain euphoria that he learned about from the internet. The patient reported a syncopal episode, one day prior to coming to the ED, which was worked up at an outside hospital. A computed tomography (CT) scan of the head done there revealed unremarkable findings. The patient was alert and oriented to time, place, and person. He complained of chills, nausea, vomiting, constipation, and abdominal cramps at the time of admission. He denied chest pain, breathing difficulty, and prior history of cardiac disease. An electrocardiogram (EKG) was done that showed right bundle branch block (RBBB), bradycardia with heart rate (HR) 51 bpm and normal QTc. Cardiac ischemia and other drug toxicities were ruled out. Sodium was 136 mEq/L and glucose was 92 mg/dL on presentation. Electrolytes and thyroid function tests were within normal range. He was admitted to the telemetry floor and placed on a continuous cardiac monitor with normal saline intravenous for hydration. Telemetry strips showed bradycardia with HR ranging from 39 to 58 bpm and RBBB. An abdominal X-Ray was obtained that showed constipation. Patient was given laxatives that helped with constipation. He was seen by cardiology to assess the EKG changes. An echocardiogram was done which was normal; the left ventricular ejection fraction was 55%. The patient was then evaluated by psychiatry and started on venlafaxine and gabapentin for mood and anxiety. He remained asymptomatic and was discharged home with a Holter monitor. The patient was advised to discontinue loperamide use and was briefed of its potential hazards upon using it in such high doses. He was referred to an addiction treatment program.

## Discussion

Loperamide is an antidiarrheal opioid that can produce life-threatening toxicity at high doses including cardiotoxicity, central nervous system (CNS) depression, respiratory depression, and urinary retention. Cardiac toxicity may present with symptoms ranging from palpitations to sudden cardiac death. Several reports suggest patients presenting with ventricular dysrhythmias, torsades de pointes, and even cardiac arrest. Reported loperamide-induced EKG disturbances include bradycardia, widening of QRS, and QTc prolongation. The ability of high-dose loperamide to block cardiac potassium and sodium channels provides the basis of EKG abnormalities and dysrhythmias [9].

Presyncope, recurrent syncope, dyspnea, and seizure-like activity have also been reported with loperamide abuse. The drug predominantly exhibits peripheral activity, low bioavailability, and poor blood-brain barrier penetration at the FDA-approved doses. However, when ingested in massive doses (40-100 times the usual dose), it can cross the blood-brain barrier and exert CNS activity similar to that of other opioids [3]. Loperamide has a relatively long half-life of 9 to 13 hours. At doses of 16 mg and higher, the half-life has been found to be as high as 41 hours. Our knowledge about drug interactions with loperamide is limited. CYP3A4 inhibitors (e.g., clarithromycin, cyclosporine, erythromycin, itraconazole, ketoconazole, verapamil, cimetidine) and CYP2C8 inhibitors (e.g., gemfibrozil, clopidogrel, grapefruit juice, amitriptyline, trimethoprim) have been reported to increase the plasma concentration of loperamide by about twofold and fourfold, respectively [10]. Inhibitors of P-gp (e.g., clarithromycin, erythromycin, ketoconazole, verapamil, quinidine, ritonavir, ranolazine,) also increase the plasma concentration of loperamide by twofold to threefold and may increase the loperamide CNS concentration by reducing the effectiveness of the blood-brain barrier [11]. Therefore, these should be used cautiously with loperamide.

Supportive care is the mainstay of management. A blood test that specifically measures loperamide levels can be done because a standard drug screening will not detect it. Naloxone has been used to reverse coma and respiratory depression. For treatment of loperamide-induced cardiotoxicity, standard advanced cardiac life support should be followed including cardioversion or defibrillation for shockable rhythms and intravenous magnesium for polymorphic ventricular tachycardia. For QRS interval widening, a trial of sodium bicarbonate is suggested [9]. Even in asymptomatic patients and drug discontinuance, physicians should consider obtaining a consultation with a medical toxicologist, promptly treat ECG abnormalities aggressively, and admit all patients for further monitoring. Also, consider treating underlying opioid use disorder and making a referral to an addiction treatment program.

## Conclusions

Health care providers should counsel patients about the cardiac risks associated with high doses of loperamide, and urge them to stick with the recommended dose. Patients with opioid use disorder should be treated with FDA-approved drugs to reduce opioid withdrawal symptoms. Health care providers should be cautious about prescribing loperamide to patients who are at risk for serious heart problems, are predisposed to QT interval prolongation, or who are already on medications known to inhibit loperamide metabolism.

In the current age of opioid epidemic in the US and around the world, there is a burgeoning rise in the abuse of loperamide for its euphoric properties and physicians must be aware of potential dangers of abusing this medication. It is vital that, at the time of admission, a review of all the OTC medications taken by patients should be done to help suspect and diagnose loperamide toxicity. Typically, a patient with loperamide abuse will have a history of opioid dependence or discontinuation of prescription opioids. It has become imperative for emergency physicians, internists, and psychiatrists to rule out loperamide abuse in this vulnerable population and identify life-threatening consequences to be treated effectively.
